# Vitamin D binding protein isoforms as candidate predictors of disease extension in childhood arthritis

**DOI:** 10.1016/j.jprot.2012.06.024

**Published:** 2012-09-18

**Authors:** David S. Gibson, Keri Newell, Alexandra N. Evans, Sorcha Finnegan, Gwen Manning, Caitriona Scaife, Catherine McAllister, Stephen R. Pennington, Mark W. Duncan, Terry L. Moore, Madeleine E. Rooney

**Affiliations:** a Arthritis Research Group, Queen's University of Belfast, Centre for Infection and Immunity, Health Sciences Building 97 Lisburn Road, Belfast, BT9 7BL, UK; b Division of Endocrinology, Metabolism and Diabetes, School of Medicine, University of Colorado Denver, 12800 E. 19th Ave., Aurora, CO 80045, USA; c Proteome Research Centre, Conway Institute for Biomolecular and Biomedical Research, School of Medicine and Medical Science, University College Dublin, Dublin D4, Ireland; d Obesity Research Center, King Saud University, P.O. Box 2925, Riyadh 11461, Saudi Arabia; e Division of Adult and Pediatric Rheumatology, Saint Louis University School of Medicine, Saint Louis, MO, USA

**Keywords:** Juvenile idiopathic arthritis, Proteomics, Synovial fluid, Vitamin D binding protein, Inflammation

## Abstract

**Introduction.:**

Juvenile idiopathic arthritis (JIA) comprises a poorly understood group of chronic autoimmune diseases with variable clinical outcomes. We investigated whether the synovial fluid (SF) proteome could distinguish a subset of patients in whom disease extends to affect a large number of joints.

**Methods.:**

SF samples from 57 patients were obtained around time of initial diagnosis of JIA, labeled with Cy dyes and separated by two-dimensional electrophoresis. Multivariate analyses were used to isolate a panel of proteins which distinguish patient subgroups. Proteins were identified using MALDI-TOF mass spectrometry with expression verified by immunochemical methods. Protein glycosylation status was confirmed by hydrophilic interaction liquid chromatography.

**Results.:**

A truncated isoform of vitamin D binding protein (VDBP) is present at significantly reduced levels in the SF of oligoarticular patients at risk of disease extension, relative to other subgroups (*p* < 0.05). Furthermore, sialylated forms of immunopurified synovial VDBP were significantly reduced in extended oligoarticular patients (*p* < 0.005).

**Conclusion.:**

Reduced conversion of VDBP to a macrophage activation factor may be used to stratify patients to determine risk of disease extension in JIA patients.

## Graphical abstract

**Figure:**
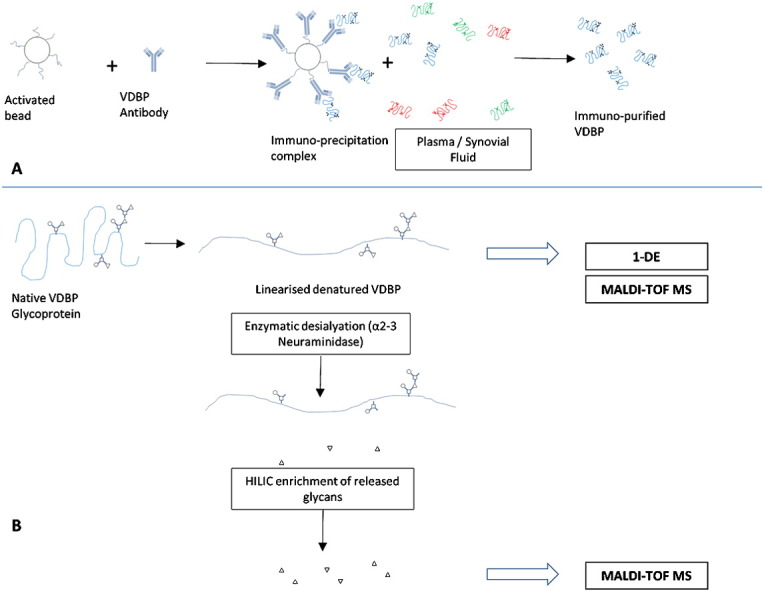

## 1 Introduction

About one in every thousand children in the UK suffers from juvenile idiopathic arthritis (JIA) [1]. JIA is a heterogeneous group of inflammatory disorders affecting the musculoskeletal system. Of the seven subsets of JIA identified according to ILAR classification [2], oligoarticular, extended oligoarticular, and polyarticular are the commonest. Adverse outcomes can present to varying degrees regardless of disease subtype [3]. In approximately 25% of children with oligoarticular JIA, over time the disease spreads to involve many joints, a condition known as extended oligoarticular disease [4]. Clinical, laboratory or radiologic parameters cannot accurately predict disease extension. Extended oligoarticular JIA is much more difficult to treat due to its characteristic resistance to second‐line therapies [5]. It is therefore important to define more sensitive markers to determine the risk of inflammation spreading to previously unaffected joints. If it was possible to identify these children earlier, more effective therapies could be instigated to prevent joint and periarticular damage.

Previous studies have suggested that measurement of a selected set of synovial fluid or plasma proteins may be used to discriminate clinically and biologically relevant JIA subgroups [6–9]. A recent study reported a significant reduction in the ratio of CD4:CD8 positive T cells with a corresponding increase in the levels of CCL5 in the synovial fluid of extended oligoarticular patients [10].

Post‐translational modifications of proteins are frequently overlooked as candidates are identified in biomarker discovery studies. However, covalent modifications of proteins by oxidation, phosphorylation or glycosylation can have profound effects on protein transport, function, stability and recognition. Growing evidence suggests a significant role for glycosylation in a range of arthritic and autoimmune disorders [11,12]. Specifically, protein glycosylation motifs affect a wide variety of innate and adaptive immunological processes including inflammation, cellular infiltration, cell communication and adhesion, and lymphocyte tolerance [13–17]. Changes in the glycosylation of proteins such as acute-phase proteins and antibodies have already been recorded in the synovial fluid and plasma of arthritis patients, but no relationship to clinical subtype or outcome has yet been established in JIA [18–22].

This study is focused on identifying protein isoforms in a de novo cohort of children with newly diagnosed JIA that will predict disease spread. The synovial fluid proteome of the persistent oligoarticular patient subgroup was compared to that of patients who show a spread after the first 6 months post diagnosis to involve five or more joints i.e., the extended-to-be oligoarticular subgroup. Novel mass spectrometry based analyses were employed to resolve protein post-translational modifications which are not apparent by conventional antibody based methods.

## 2 Materials and methods

### 2.1 Patients

Fifty-seven patients with newly diagnosed *untreated* JIA according to International League Against Rheumatism criteria entered this study and were followed for 1 year. At the time of initial sampling there were 34 children with oligoarticular arthritis, 18 with polyarticular arthritis (16 rheumatoid factor negative) and 5 with psoriatic or enthesitis related arthritis. Patient data shown in Table 1 refers to clinical findings at the time of joint aspiration and biopsy i.e. at initial presentation before disease extension. Disease extension was defined as 5 or more joints involved after 6 months from disease commencement. At 1 year, 8 oligoarticular cases had been reclassified as having extended oligoarticular JIA.

Patients were examined by a consultant rheumatologist (M.E.R.) who confirmed their diagnosis. For the purposes of this study, only initial synovial fluids from children with disease duration of less than 1 year and steroid and DMARD naive were included. Arthrocentesis and subsequent joint steroid injection were performed according to clinical need.

Clinical details recorded included subtype of JIA, age, sex, disease duration, erythrocyte sedimentation rate (ESR) and C-reactive protein (CRP). Treatments applied after samples were drawn are also listed. Local inflammation was defined as both joint swelling and pain on physical examination. All SFs were aspirated using an aseptic technique; plasma was obtained at the same visit. Samples were immediately centrifuged (5000 *g*, 15 min, 4 °C), aliquoted and stored (− 80 °C) for at least 1 year to allow for clinical reclassification. Medical Ethics Committee approval was obtained for this study at Green Park Healthcare Trust and patient assent and parent informed consent given (ORECNI 408/03).

### 2.2 Sample preparation

Samples were dialyzed (overnight, 4 °C, distilled water) to remove salts using a 3.5 kDa cutoff Slide-A-Lyzer cassette (Pierce Biotechnology, Inc., Rockford, IL). Each sample was snap frozen in liquid nitrogen and lyophilized overnight on a freeze-dryer (Martin Christ GmbH, Osterode am Harz, Germany). Samples were rehydrated in sample rehydration buffer (8 M Urea, 2% CHAPS and 0.002% bromophenol blue; Invitrogen Ltd., Paisley, UK). Protein concentrations were measured using the PlusOne 2-D Quant kit according to the manufacturer's guidelines (GE Healthcare, Bucks, UK).

### 2.3 Difference in-gel electrophoresis (DIGE)

DIGE was performed at room temperature with Ettan IEF and vertical gel systems and associated power supply, strips, gels and reagents according to the manufacturer's guidelines (GE Healthcare, Bucks, UK) as described before [9,22]. Each synovial fluid and plasma sample was minimally labeled with Cy5 and Cy3 fluorescent dyes and an internal pooled standard (Cy2) sample was included according to the manufacturer's recommendations. 50 μg of each Cy5 and Cy3 labeled sample and Cy2 labeled standard was combined and resuspended in an equal volume of sample buffer (8 M Urea, 130 mM DTT, 4% (w/v) CHAPS, 2% (v/v) Pharmalyte 4–7). 24 cm Immobiline DryStrip pH 4–7 linear immobilized pH gradient (IPG) strips were rehydrated overnight with the relevant sample mixes. First-dimension separation of proteins by isoelectric focusing (IEF) was performed for a total of 75,000 Vh (2 mA/5 W limit per strip) including a final 8000 V step for 1 h to obtain improved resolution. After IEF, the strips were equilibrated first in 1% (w/v) dithiothreitol and then 2.5% (w/v) iodoacetamide. IPG strips were each laid into single well 12% PAGE gels and sealed in place with 1% agarose (w/v) in running buffer (25 mM Tris, 192 mM glycine, 0.1% (w/v) SDS and bromophenol blue). The second dimension separation was undertaken at 0.75 W/gel for 19 h. A preparative gel loaded with 500 μg of unlabeled sample was silver-stained with mass spectrometry compatible reagents for spot excision (Pierce Biotechnology, Inc., Rockford, IL, USA).

### 2.4 Image and cluster analysis

Pre-labeled proteins were visualized using a Typhoon 9410 imager (GE Healthcare, Bucks, UK). Gel image analysis was performed with Progenesis Samespots software (version 2.0, build 2644.18003; Nonlinear Dynamics Ltd., Newcastle upon Tyne, UK). All gel images were aligned to a reference gel and the same spot outlines were overlaid onto all images to ensure no data were omitted. The normalized volume (NV) for each spot on each gel was calculated from the Cy3 (or Cy5) to Cy2 spot volume ratio. Log transformation of the spot volumes was used to generate normally distributed data. Log normalized volume (LNV) was used to compare spot abundance. Differential spot analysis was performed on aggregate ‘master’ gels of the patient subgroups. Each comparison was filtered to find spots (a) with a *p*-value < 0.05 for the unpaired *t* test and (b) having a greater than 1.5-fold change in average LNV expression between the groups.

Expression data were analyzed using Epclust, a generic data clustering, visualization, and analysis tool (http://www.bioinf.ebc.ee/EP/EP/EPCLUST/). Hierarchical analysis reordered protein expression patterns in an agglomerative fashion, using the weighted pair-group average (WPGMA) clustering procedure. Euclidean ranked correlation was the similarity measure used to group or separate the expression data. A heat map was produced accompanied by a dendrogram depicting the extent of similarity between the different groups in the samples.

### 2.5 Mass spectrometry identification and verification

Protein spots were excised from silver-stained 2DE gels and digested according to the protocol described previously [8]. Briefly, the gel spots were washed, reduced and alkylated, then dehydrated with acetonitrile. The proteins were digested overnight with trypsin (Promega, Southhampton, UK; modified trypsin, 37 °C) and the resulting peptides concentrated on a ZipTip micro purification column and eluted onto an anchor chip target for analysis (4800 MALDI-TOF/TOF mass spectrometer; Applied Biosystems, Warrington, UK). Mass analysis was performed in the positive ion reflector mode. Some of the peptides from each digest were analyzed in MS/MS mode to obtain partial peptide sequence data. Mass spectra were acquired in the 800–4000 *m*/*z* scan range (Table 2). The mass accuracy was calibrated to within 50 ppm using calibration standards (a mix over 900–3700 *m*/*z* from Applied Biosystems). To identify proteins, MS data were used to query the non-redundant and validated sequence database (Uni-Prot 2009.09.23; contained 522,019 entries) using Mascot (version 2.2.03).

Database search parameters were: (i) trypsin cleaves on the C-terminal side of K and R residues unless the next residue is P, (ii) no fixed modifications, (iii) carbamidomethyl (C) and oxidation (M) variable modifications, (iv) up to 1 missed cleavage permitted with no fixed modifications, (v) peptide tolerance set at 100 ppm for the precursor ions, and (vi) a 0.25 Da mass tolerance for the fragment ions. The acceptance criteria for PMF based identifications was a minimum Mascot score of 50, using a 95% confidence interval threshold (*p* < 0.05). The peptide ions identified in this study by MALDI-TOF and further validated by collision induced dissociation (CID) MS/MS analysis were independently matched to single protein entries in the database.

### 2.6 Immunohistochemistry and ELISA

Immunohistochemistry was performed on synovial membrane biopsies to confirm tissue expression. Synovial membrane tissues were obtained from each patient by needle biopsy, coated in OCT compound, snap frozen in liquid nitrogen and stored at − 80 °C. Cryostat sections of 7 μm were cut (Leica CM 1900; Meyer Instruments, Inc.), acetone fixed, air-dried and then rehydrated. Endogenous peroxidase was blocked, sections rinsed and probed with VDBP primary antibody, Factor VIII or isotype matched antibody (negative control). Biotinylated *Sambucus nigra* lectin was used to probe for sialic acid residues (Vector laboratories Inc., Burlingame, CA, USA). Tissue sections were incubated with the Envision + Dual link system sHRP (DAKO A/S, Glostrup, Denmark) or streptavidin HRP polymer (Sigma-Aldrich Inc., St. Louis, MO, USA). Again, sections were washed, stained with DAB solution, rinsed and counterstained in Mayer's hematoxylin. Sections were washed, dehydrated, and air-dried. Sections were cover-slipped and imaged with an Olympus BX41 light microscope and JVC 3CCD camera (Olympus Ltd., Essex, UK).

Enzyme-linked immunosorbant assays (ELISA) for C-reactive protein (CRP) and VDBP (R&D Systems, Minneapolis, MN, USA) were performed according to the manufacturer's guidelines on both synovial fluid and plasma from the study cohort. ELISA plates were read on a Multiscan MCC/340 plate reader at 450 nm (Thermo Labsystems, Helsinki, Finland).

### 2.7 Immunoprecipitation, desiaylation and mass spectrometry analysis

VDBP polyclonal antibody (DAKO A/S, Glostrup, Denmark) was immobilized on Direct IP© agarose beads (Thermo Pierce Scientific, Rockford, IL, USA) to form the immune complex. Synovial fluids from all study patients were incubated with the immune complex overnight at 4 °C with end to end mixing. The complex was washed according to the manufacturer instructions and bound VDBP was dissociated with a minimal volume of low pH elution buffer. Immunopurified VDBP samples were lyophilized and resolubilized in 0.1% RapidGest SF (Waters Technologies Corporation, Millford, MA, USA) in 50 mM NH_4_HCO_3_, reduced with dithiothreitol and alkylated with iodoacetamide.

The linearized VDBP was then split for three separate analyses: (i) one dimensional gel electrophoresis and (ii) MALDI-TOF mass spectrometry, of the intact protein, and (iii) enzymatic removal and mass spectrometry of sialic acid residues. A Criterion gel tank and 4–20% TGX precast gel were run according to manufacturer's guidelines (Biorad Laboratories Inc., Hercules, CA, USA). A purified form of human VDBP was run with IP samples as a positive control (2 μg) (Athens Research & Technology Inc., Athens, GA, USA). Once the gels were run, proteins were fixed (with 7% glacial acetic acid, 30% methanol) and visualized with GelCode Blue coomassie dye reagent (Thermo Pierce Scientific, Rockford, IL, USA). Immunopurified samples of VDBP were resuspended in 80% acetonitrile, 0.1% trifluoroacetic acid and spotted in duplicate onto a MALDI target plate with sinnapinnic acid matrix. Intact proteins were analyzed using a Voyager STR + MALDI-TOF mass spectrometer in linear mode (Applied Biosystems Corporation, Carlsbad, CA, USA). A high molecular weight mixture was used for calibration (Bruker Daltronics Inc., Billerica, MA, USA).

Equal quantities of denatured VDBP were treated with α2-3 neuraminidase (sialidase) overnight at 4 °C to remove sialic acid residues (New England Biolabs Inc., Ipswich, MA, USA). Released glycans were enriched and separated from protein by MassPREP™ hydrophilic interaction liquid chromatography (HILIC) mElution plate (Waters Technologies Corporation, Millford, MA, USA). Glycan samples were resuspended in ethanol with 2,5-dihydroxybenzoic acid (DHB) matrix and spotted in duplicate onto a MALDI target plate. Glycans were analyzed in triplicate using a Voyager STR + MALDI-TOF mass spectrometer in reflector mode. A low molecular weight mixture was used for instrument calibration (Bruker Daltronics Inc., Billerica, MA, USA). Progenesis MALDI software v1.4 was used to normalize, remove noise and align MALDI-TOF spectra and accurately assess ion masses (Nonlinear Dynamics Ltd, Newcastle upon Tyne, UK). Peaks were normalized by total ion current and normalized peak height was used as the statistics measure.

### 2.8 Statistical analysis

Significant differences between DIGE normalized spot volumes of the study subgroups were calculated using the unpaired Student's *t*-test (within Progenesis Samespots software); MALDI normalized peak height data was analyzed by ANOVA (within Progenesis MALDI software). DIGE, MALDI and ELISA data were analyzed using GraphPad Prism (version 5.03; GraphPad Software Inc., La Jolla, CA, USA) to construct receiver operator characteristic curves.

## 3 Results

### 3.1 Synovial fluid proteome and differentially abundant proteins

**Fig. 1:**
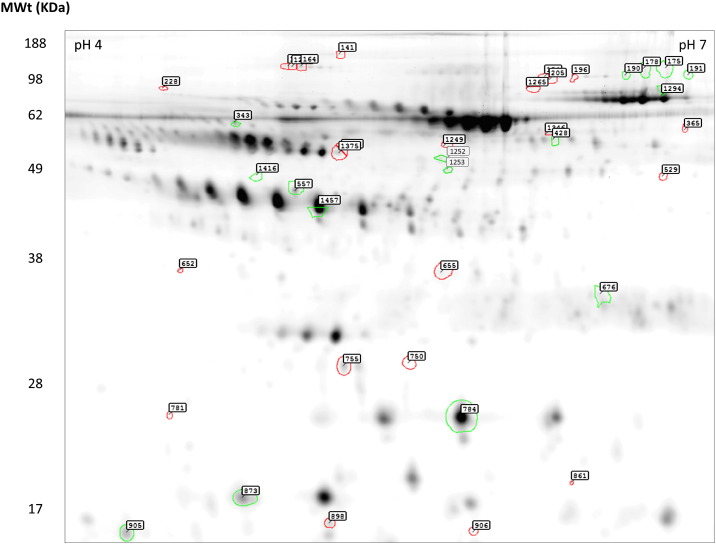
Fluorescence difference in-gel electrophoresis (DIGE) of synovial fluid. DIGE reveals 1300 spots per gel within the pH 4–7 range for synovial fluid (SF). Spot filtering on aggregate ‘master’ gels reveals 426 protein spots which are consistently expressed in synovial fluid across patient subgroups. A series of 39 proteins that changed 50% or more in SF in later extended oligoarticular compared to persistent oligoarticular patients are encircled and numbered above. The direction of the expression change is indicated such that spots encircled green denote decreased proteins whereas red are increased relative to articular patients. Spot numbers correlate to the clusters observed in the data after hierarchical cluster analysis, shown in Fig. 2. 68 proteins spots were cut from replicate preparative gels and 36 of these identified by MALDI-TOF analysis.

**Fig. 2:**
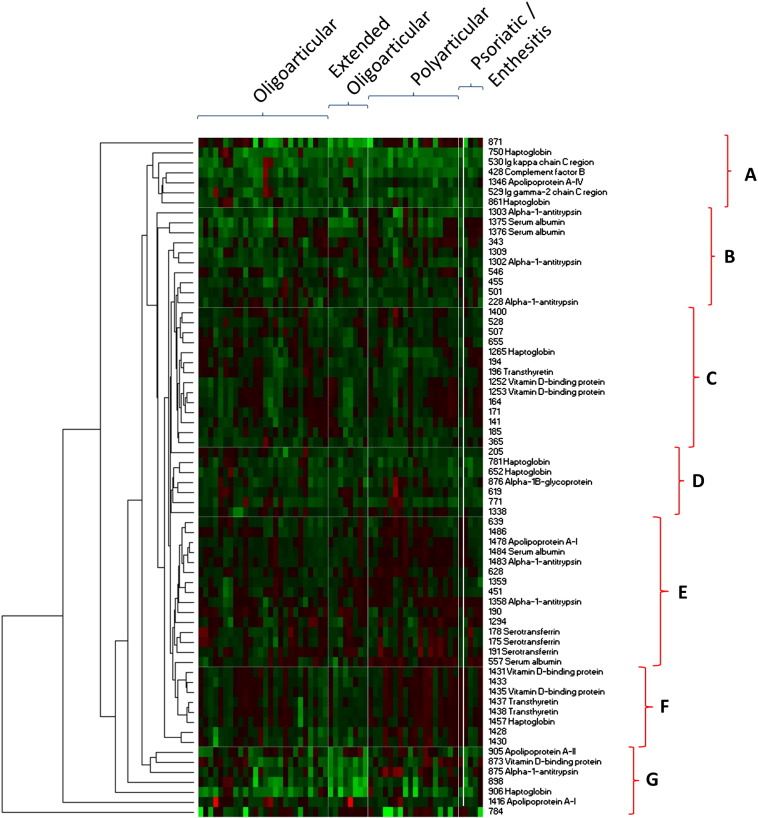
Heatmap analysis of synovial proteins differentially expressed across patient subgroups. The inter-individual variation in 68 preselected proteins over or under expressed 1.5 fold between patient subgroups is illustrated in heat map form. The protein expression data was reordered by hierarchical cluster analysis (HCA) using Euclidean distance correlation (WPGMA), revealing distinguishing expression patterns. The main clusters of proteins are highlighted (A–G). Each patient sample is represented by a single column, whereas each row represents a single protein spot. The location of 36 proteins which changed in oligoarticular patients who later become extended were identified from preparative gel by MALDI-TOF mass spectrometry are shown in Fig. 1.

Synovial fluid samples from 57 JIA patients (Table 1) were resolved on 2D gels to determine protein expression profiles. It is apparent that several proteins formed a series of charge trains (Fig. 1). This characteristic pattern of high-abundance proteins is consistent with previous work by other laboratories and our own [23,24]. Approximately 1300 spots per synovial fluid gel image were detected and matched across patients. Spot filtering on ‘master’ gels revealed 558 protein spots which are differentially expressed in synovial fluid across patient subgroups. Attention was focused on a series of 68 synovial fluid proteins (listed in Fig. 2) which displayed a minimal 1.5-fold difference in pairwise comparisons between subgroups.

### 3.2 Discriminatory protein clustering, inter-subgroup variation and protein identification

A heatmap was constructed with hierarchical cluster analysis to visualize inter-individual expression patterns of the 68 filtered protein spots (Fig. 2). Euclidean ranked correlation delineates seven distinctive clusters (A–G). Clusters C, F and G comprise proteins consistently overexpressed (red) in both oligoarticular and polyarticular patients relative to those patients with disease extension, whereas protein levels in clusters A, B and D are raised intermittently across all subgroups. Cluster E contains proteins which are principally amplified in polyarticular and extended-to-be oligoarticular patients.

Amongst the inter-subgroup differences which were significant at the 5% level, protein spot 873 stands out (Table 2). It resides within the distinctive cluster G of proteins that are markedly suppressed in patients exhibiting disease extension. Spot 873, identified as an isoform of vitamin D binding protein (VDBP) (see below), was decreased 8 fold and 4 fold in extended-to‐be oligoarticular patients relative to persistent oligoarticular and polyarticular patients, respectively. These differences are significant (*p* = 0.05 and *p* = 0.03, respectively). Two additional VDBP isoforms present as protein spots of higher molecular mass (spots 1252 and 1253) showed about a 2 fold decrease in disease extended-to-be oligoarticular patients (*p* = 0.01).

**Fig. 3:**
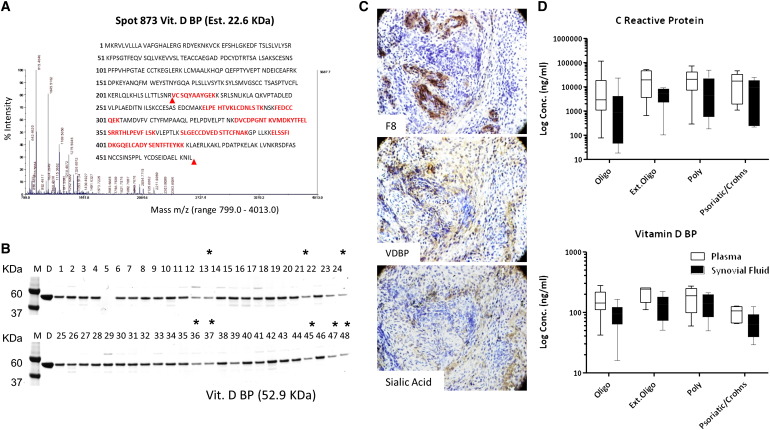
Verification of protein expression and localization in synovial fluid and membrane. A. Representative MALDI-TOF mass spectra of spot 873 identified as vitamin D-binding protein. The matched peptide sequences are underlined within the sequence of vitamin D-binding protein. An approximate molecular weight of the protein cleavage product (between the arrows) is calculated from the amino acid sequence, in line with DIGE estimate. B. Immunoprecipitation of VDBP from synovial fluids of n = 48 representative JIA patients (indicated by number in lanes 1–48; M — molecular weight marker; D — purified human VDBP positive control). Band densities of vitamin D binding protein concur with protein expression levels measured by DIGE (extended-to-be oligoarticular patients in lanes 13, 21, 24, 36, 37, 45, 47, 48). C. Representative immunohistochemistry of neighboring sections of synovial membrane from a polyarticular patient, all captured at 10 × magnification. Vitamin D binding protein expression is perivascular in nature. D. ELISA quantification of c-reactive protein and vitamin D binding protein concentration in initial plasma and synovial samples taken from the whole study cohort. Error bars on the box-whisker plots represent range between maximum and minimum values; center line represents median.

Trypsin digestion and mass spectrometry (MS) followed by MS/MS of spots 1252, 1253, 1431 and 1435 produced peptides that aligned with the majority of VDBP amino acid sequence (Supplementary Fig. 1). Since a number of differentially expressed VDBP spots were extracted from disparate sites on the 2DE gel, these proteins likely represent isoforms with subtle pI or molecular weight differences due to post-translational events. However, digestion of spot 873 showed peptides that aligned with a reduced portion of the parent protein sequence between amino acids 218 and 420 (Fig. 3A). This data raises the possibility of a low molecular weight (approx. 23 kDa), possibly truncated isoform of VDBP distinct from the complete protein (54.3 kDa).

Several other proteins including serum albumin (1375), complement factor B (428), alpha-1-antitrypsin (228) and several haptoglobin isoforms (750, 1265) were also substantially reduced in extended-to be oligoarticular patients. Conversely, apolipoproteins A-I (1416) and A-II (905), and to a lesser extent other haptoglobin isoforms (906, 1457), were increased in the same subgroup of extended patients.

### 3.3 Verification of immunoreactive VDBP expression

Immunoprecipitation of synovial fluid samples and immunohistochemistry of synovial membranes from a representative subset of patients were used to independently validate the identification and expression patterns of VDBP. Immunopurified and linearized VDBP band densities are consistent with DIGE spot intensities of spots 873, 1252 and 1253 (Fig. 3B). A major band at approximately 52.9 kDa is expressed heterogeneously within patient subgroups, but nonetheless diminished within the extended-to-be oligoarticular subgroup.

Synovial membrane sections probed with the same VDBP antibody displayed a disparate stain distribution characteristic of perivascular expression at distinct sites within the sublining layer of the tissue (Fig. 3C). Sialic acid glycosylation motifs on synovial tissue proteins produced a similar pattern.

A VDBP ELISA was used to measure immunoreactive VDBP in each of the 57 individual JIA patient's plasma and synovial fluid samples (Fig. 3D). Both immunoreactive VDBP and CRP were higher in plasma than in synovial fluid. Immunoreactive VDBP levels ranged from 42.0 to 277.0 ng/ml in plasma vs. 16.0 to 213.0 ng/ml in synovial fluid. CRP concentrations varied over a wider interval: i.e., 78.0–115,149.0 ng/ml in plasma vs. 18.0–49,486.0 ng/ml in synovial fluid. VDBP and CRP were markedly elevated in the plasma of both the extended-to-be oligoarticular and polyarticular patient groups.

### 3.4 Verification of immunopurified VDBP isoform mass variance and sialic acid modification

**Fig. 4:**
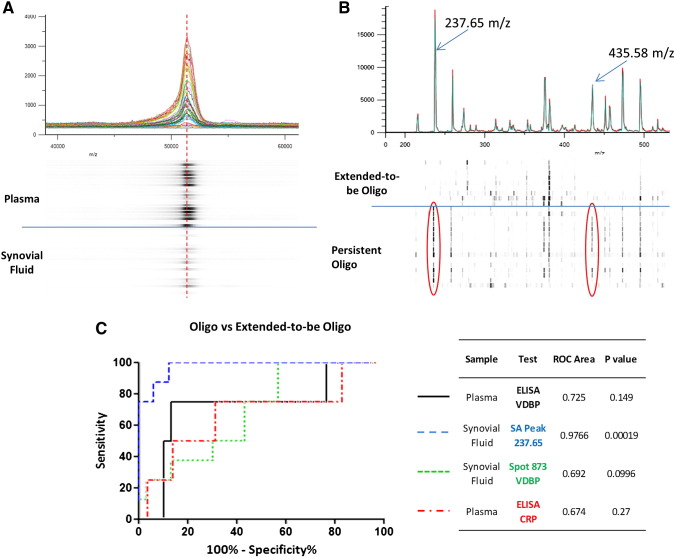
MALDI-TOF mass spectrometry of intact immunopurified VDBP and enzymatically released glycans diagnostic capacity. A. MALDI-TOF mass spectrometry of immunoprecipitated and linearized VDBP from a representative pool of study patients. The peak intensity represented in as an individual ‘densitometry’ lane below the chart, reflects higher plasma concentrations of VDBP. The majority of intact forms of VDBP from plasma and synovial fluid have a peak mass of 52,900 *m*/*z* (represented by the red dashed line). A peak shift towards the right resulting in a ‘shoulder’ is apparent, indicating higher mass variants or isoforms of VDBP are also present in both fluids. B. MALDI-TOF mass spectrometry of HILIC enriched glycan released by α2-3 neuraminidase digest from immunopurified VDBP from the synovial fluid of oligoarticular patients. Glycan peaks at 237.65 *m*/*z* and 435.58 *m*/*z*, representing release of sialic acid residues, are indicated by the red encircled bands within individual patient ‘densitometry’ lanes. There is a significant 72.4 fold difference in the normalized 237.65 *m*/*z* peak heights between persistent and extended-to-be oligoarticular patients, signifying few sialic acid modifications detected in the VDBP taken from the latter patients (*p* = 0.0014). C. Receiver operator characteristic curves to test the sensitivity and specificity of VDBP (black line) and CRP (red line) ELISA concentration values and DIGE derived normalized volumes for spot 873 (green line) and sialic acid (peak 237.65 *m*/*z*; blue line) released from VDBP to discern patients at risk of disease spread. Area under the curve and *p* values for each test are also included in the accompanying table.

MALDI-TOF mass spectrometry of immunoprecipitated and linearized VDBP from study patients reveals a major peak with mass 52,900 *m*/*z* in line with estimates from the gel electrophoresis and elevated plasma concentrations in agreement with ELISA data (Fig. 4A). However, a peak shift resulting in a ‘shoulder’ is apparent, indicating higher mass variants or isoforms of VDBP are also present in both fluids. Hydrophobic interaction liquid chromatography (HILIC) enriched glycans, released by α2-3 neuraminidase digest of immunopurified SF VDBP from oligoarticular patients, were analyzed by MALDI-TOF mass spectrometry. Glycan peaks representing the release of sialic acid residues from the peptide backbone of VDBP are evident at 237.65 *m*/*z* and 435.58 *m*/*z* (Fig. 4B). The normalized peak heights were significantly elevated in persistent oligoarticular patients, signifying that fewer terminal sialic acid modifications were present on VDBP from extended-to-be patients. The 237.65 *m*/*z* peak height was 72.39 fold higher (*p* = 0.00019) and 435.58 *m*/*z* was 5.58 higher (*p* = 0.00593) in persistent oligo patients.

### 3.5 Assessment of VDBP isoforms as predictive tools

The diagnostic specificity and sensitivity of immunoreactive VDBP and CRP (determined by ELISA) to predict disease extension were compared to that of VDBP isoform from spot 873 (using DIGE spot volumes) and the normalized peak height of sialic acid (peak 237.65 *m*/*z*) enzymatically released from immunopurified VDBP (Fig. 4C). Receiver operator characteristic curves indicate that the VDBP sialic acid motifs measured by MALDI-TOF give the best specificity to identify at risk individuals (AUC 0.976; *p* = 0.00019). This compares favorably to VDBP (AUC = 0.725), spot 873 (AUC = 0.692) and CRP (AUC = 0.674). Sensitivity to identify at risk patients is marginally improved with spot 873, relative to immunoreactive VDBP. Furthermore, the discriminatory power of spot 873 was closer to significance at *p* = 0.099 than either immunoreactive VDBP (*p* = 0.149) or CRP (*p* = 0.270).

## 4 Discussion

Conventional measures of the acute phase response, including CRP, were recorded within this study at similar levels within in oligo and extended-to-be oligoarticular groups. These markers do not aid in the clinical differentiation of patients who will likely develop a range of adverse outcomes. However, significantly higher levels (of CRP) were observed in the polyarticular patients signifying the increased disease activity experienced at an early stage by polyarticular patients.

In contrast high resolution proteomic strategies can be used to identify and quantify proteins and importantly characterize the distinct modifications associated with a particular disease subset. Recently, other groups have successfully adopted a proteomic platform to identify biomarkers which discern JIA subgroups building on our own preliminary studies [6,9,25]. Rosenkranz et al. identified a subset of the synovial proteome which could distinguish between oligoarticular, polyarticular and systemic forms of JIA. Haptoglobin emerged as a particularly strong candidate biomarker. Ling et al. identified a panel of seven plasma proteins which can discriminate patients at risk from an impending disease flare with greater reliability than CRP or ESR. These results suggest that for each clinical outcome/subtype a proteomic profile exists and which has diagnostic or prognostic potential.

This study documents a constellation of distinct vitamin D binding proteins (VDBPs) with unique pI/MWt coordinates produced in synovial fluid. Notably, in the extended-to-be oligoarticular patient subgroup, a low molecular weight species of VDBP is significantly reduced in the joint. This may reflect increased degradation or represent a novel post-translational truncation in at risk patients. Equally, the elevated levels of immune-reactive VDBP, measured by ELISA, in the plasma of extended-to-be oligoarticular patients is a further indication of an altered expression pattern. This data may seem incongruous until one considers the technical differences of the analyses. Since the ELISA assay relies on polyclonal capture and recognition of VDBP it is unlikely to distinguish particular isoforms of the complete protein. Low molecular weight isoforms may be missing the antibody detected epitopes and therefore ‘overlooked’ by ELISA determination. Likewise the significantly reduced glycosylation of SF VDBP from extended-to-be oligoarticular patients would also go undetected by ELISA. This illustrates the added benefit of using a proteomic platform such as DIGE which can discern distinct protein isoforms and highlights the importance of verifying protein micro-heterogeneity across patient populations when advancing biomarker candidates to clinical validation [25].

VDBP (also known as Gc globulin) is an albumin-like protein that requires cell surface binding to mediate some of its functions [26]. Its reported biological roles include vitamin D metabolite transport, fatty acid transport, actin sequestration, complement C5a chemotactic factor binding and inhibition of angiogenesis [27–30]. However, of special relevance to the current study, VDBP has received much attention in recent years as a precursor of the potent macrophage activation factor Gc-MAF which can in turn release pro-inflammatory cytokines at affected sites. Two of the best characterized forms of VDBP, Gc1s and Gc1f, can have an O-linked trisaccharide coupled to a threonine residue at position 420 (T420) in the amino acid sequence. To date, 120 variant isoforms have been reported [30–32]. Studies suggest that when VDBP is subjected to sequential B cell β-galactosidase and T cell sialidase treatment, this generates GcMAF by converting the trisaccharide at T420 to a single sialic acid residue (GalNAc) [32–34]. Substitution for a lysine at this position in an alternate form Gc2 prevents this isoform from being glycosylated at this position. In addition to the subtype related levels of VDBP noted in this study cohort, it is compelling to suggest that the differential modification of the protein observed may also be important in the pathology of JIA. Studies of biologic drug efficacy in the rheumatic disorders RA and JIA report increased levels of systemic VDBP and apolipoprotein A-I in successful responders, suggesting a shift toward ‘normal’ macrophage activation levels [35,36]. Furthermore, VDBP has been isolated from multiple sclerosis patients suffering from a relapsing remitting form of the disease, where it is thought to play a role in T cell activation and differentiation [37]. On reflection, JIA patients who have reduced levels of most VDBP isoforms could have a disrupted macrophage activation system at the joint level which increases the chances of disease spread to distant joints by an unknown pathway. The reduced levels of sialic acid residues observed in the VDBP of extended-to-be oligoarticular patients adds further weight to this hypothesis. Similarly, reduced levels of fucosylated epitopes on an unidentified 26 kDa protein have been recorded in JIA and RA patients [18].

Independent studies suggest that along with VDBP isoforms, haptoglobin and apolipoprotein A‐II could form a composite biomarker test with greater predictive strength [6,9]. In this study extended-to-be oligoarticular patients display a general trend toward reductions in select isoforms of haptoglobin, alpha-1-antitrypsin and complement factor B relative to the other two patient subgroups. Haptoglobin has been shown to be involved in angiogenesis, tissue remodeling and cell migration [38], whereas complement activation in synovial fluid may influence phagocyte migration during joint inflammation [39,40]. Thus these host-response proteins could be involved in the spread of inflammation to previously unaffected joints.

The significant drop in the levels of truncated and glycosylated isoforms of VDBP is intriguing and suggests an altered synovial turnover of complete ‘parent’ VDBP in JIA patients at risk of disease extension. These modifications likely have a dramatic effect on the protein's function and may play a significant role in the pathology of early disease in JIA. Accordingly, changing levels of protein modification may represent a better biomarker than changes in the protein's expression levels alone. Indeed, the sensitivity and specificity observed likely stems from the combined measures of protein and glycosylation levels. The reliability of outcome prediction will evidently require further validation in independent cohorts and such a technically intricate assay would need to be converted to a more clinically robust and user friendly platform. We envision that a multiplexed protein test capable of resolving disease specific modifications could assist in predicting the disease evolution, enabling earlier appropriate intervention, and thus reducing pain, disability and joint damage.

## Abbreviations

AUC: area under curve
C3c: complement component 3c
CCD: charge coupled device
CCL5: chemokine (C-C motif) ligand 5
CD4/CD8: cluster of differentiation 4 or 8
CHAPS: 3‐((3-cholamidopropyl) dimethylammonio)-1-propanesulfonic acid
CRP: C-reactive protein
CID: collision induced dissociation
2DE: two-dimensional gel electrophoresis
DAB: diaminobenzidene
DIGE: fluorescent difference in-gel electrophoresis
DTT: dithiothreitol
ELISA: enzyme-linked immunosorbant assay
ESR: erythrocyte sedimentation rate
GcMAF: group-specific component macrophage activation factor
IEF: isoelectric focusing
IPG: immobilized pH gradient
JIA: juvenile idiopathic arthritis
K: lysine
kDa: kilodalton
LNV: log normalized volume
mA: milliamps
MALDI-TOF: matrix assisted laser desorption ionization-time of flight
mM: millimoles
MS: mass spectrometry
ORECNI: Office of the Research Ethics Committee Northern Ireland
PAGE: polyacrylamide gel electrophoresis
pH: power of hydrogen
μg: microgram
*m*/*z*: mass to charge ratio
ppm: parts per million
PMF: peptide mass fingerprint
PVDF: polyvinylidene fluoride
nm: nanometre
R: arginine
ROC: receiver operator characteristic
SDS: sodium dodecyl sulfate
SF: synovial fluid
sHRP: streptavidin horseradish peroxidise
UPMGA: unweighted pair-group average
VDBP: vitamin D binding protein
Vh: volt hours
W: watts

## Author contributions

DSG designed and carried out the proteomic studies and drafted the manuscript and performed statistical analysis. KN and AE participated in the image analysis of DIGE gels. DSG and SF carried out the immunoassays, 1-DE gel analysis and immunohistochemistry. CMcC participated in the collation of patient data. SP, GM, SP and MD contributed to the mass spectrometry analysis of protein candidates. MR conceived the patient study, and participated in the experimental design and coordination and helped to draft the manuscript. TM contributed patient samples and helped conceive the patient study. All authors read and approved the final manuscript.

## Figures and Tables

**Table 1:** Patient demographics and laboratory characteristics.

Characteristic	Subtype				Total
Oligoarticular	Extended-to-be oligoarticular	Polyarticular	Psoraitic/enthesitis
(n = 26)	(n = 8)	(n = 18)	(n = 5)	(n = 57)
Sex, no. (females)	21	7	15	2	45
Age at time of biopsy (year)	5.6 (4.4)	6.1 (4.5)	9.9 (5.0)	12.8 (3.1)	7.7 (5.1)
Disease duration (months)	6.2 (4.3)	7.4 (7.5)	7.4 (7.2)	9.0 (7.9)	7.0 (6.0)
Swollen joint count	2.3 (1.2)	3.0 (0.9)	10.9 (9.4)	–	4.5 (5.5)
Neutrophils (× 10^8^ cells/L)	4.7 (2.4)	5.9 (1.7)	5.4 (1.8)	6.7 (1.4)	5.3 (2.1)
Lymphocytes (× 10^8^ cells/L)	3.7 (1.7)	4.5 (2.1)	3.0 (1.7)	2.4 (1.1)	3.5 (1.8)
Monocytes (× 10^8^ cells/L)	0.8 (0.3)	1.0 (0.3)	0.8 (0.4)	0.7 (0.3)	0.8 (0.4)
ESR (mm/h)	16.3 (15.0)	36.5 (32.7)	38.6 (35.0)	48.0 (22.8)	29.8 (28.4)
C reactive protein (mg/L)	8.5 (14.5)	11.4 (7.8)	30.9 (43.2)	44.0 (44.2)	20.1 (32.2)
Rheumatoid factor status					
+ ve	0	0	2	0	2
− ve	26	8	16	5	55
Antinuclear antibody status					
+ ve	12	5	9	1	27
− ve	14	3	9	4	30

Clinical and demographic characteristics of the study subjects at presentation. Values are the mean ± standard deviation or the number of subjects. CRP = C-reactive protein and ESR = erythrocyte sedimentation rate.

**Table 2:** Mass spectrometry of differentially expressed proteins.

Cluster	Spot no.	Fold difference between subgroups (unpaired *t*-test with *p* < 0.05 highlighted)						Protein name	UniProt accession	Mascot score	Total ion score	Sequence coverage (%)	Matched peptides	Verification CID ions
Oligo vs ext. oligo	*p*	Oligo vs poly	*p*	Ext. oligo vs poly	*p*
A	428	− 6.32	0.30	− 6.23	0.30	− 1.02	0.95	Complement factor B	CFAB_HUMAN	69	62	4	4	1
529	− 2.60	0.31	− 4.69	0.19	1.80	0.30	Ig gamma-2 chain C region	IGHG2_HUMAN	191	146	18	8	3
530	1.20	0.86	− 8.52	0.14	10.20	0.33	Ig kappa chain C region	IGKC_HUMAN	225	195	34	2	2
750	− 7.37	0.14	− 9.96	0.13	1.35	0.31	Haptoglobin	HPT_HUMAN	244	212	16	7	6
861	− 3.20	0.11	− 1.82	0.35	− 1.75	0.38	Haptoglobin	HPT_HUMAN	368	289	28	12	7
1346	− 1.98	0.31	− 2.09	0.28	1.06	0.86	Apolipoprotein A-IV	APOA4_HUMAN	108	50	27	11	2
B	228	− 2.25	**0.002**	− 1.95	**0.02**	− 1.15	0.59	Alpha-1-antitrypsin	A1AT_HUMAN	172	118	22	10	4
1302	− 1.46	0.36	− 2.65	**0.05**	1.82	0.15	Alpha-1-antitrypsin	A1AT_HUMAN	223	189	17	8	3
1303	− 1.49	0.42	− 3.32	0.06	2.23	0.09	Alpha-1-antitrypsin	A1AT_HUMAN	293	248	16	9	5
1375	− 4.30	**0.01**	1.41	0.61	− 6.07	0.14	Serum albumin	ALBU_HUMAN	259	178	21	13	5
1376	− 2.93	**0.01**	1.28	0.67	− 3.74	0.15	Serum albumin	ALBU_HUMAN	443	326	29	18	7
C	196	− 1.61	0.08	− 1.88	**0.02**	1.17	0.48	Transthyretin	TTHY_HUMAN	493	417	72	8	4
1252	− 2.25	**0.01**	− 1.06	0.82	− 2.12	**0.01**	Vitamin D-binding protein	VTDB_HUMAN	283	222	21	11	5
1253	− 1.86	**0.01**	1.20	0.40	− 2.24	**0.01**	Vitamin D-binding protein	VTDB_HUMAN	761	640	36	16	10
1265	− 1.75	**0.04**	− 1.53	0.12	− 1.14	0.65	Haptoglobin	HPT_HUMAN	599	515	25	12	7
D	652	− 2.09	0.20	− 2.01	0.22	− 1.04	0.94	Haptoglobin	HPT_HUMAN	137	101	9	4	3
781	− 2.48	**0.01**	− 2.54	**0.01**	1.03	0.93	Haptoglobin	HPT_PONAB	166	146	14	5	2
876	1.07	0.88	1.79	0.45	− 1.67	0.51	Alpha-1B-glycoprotein	A1BG_HUMAN	106	80	13	6	1
E	175	2.23	0.14	2.21	0.10	1.01	0.99	Serotransferrin	TRFE_HUMAN	627	425	34	26	9
178	3.22	0.09	1.12	0.77	2.88	0.10	Serotransferrin	TRFE_HUMAN	628	468	22	18	11
191	3.19	0.09	2.83	0.06	1.13	0.80	Serotransferrin	TRFE_HUMAN	495	393	22	19	10
557	2.05	0.06	2.36	**0.0003**	− 1.15	0.58	Serum albumin	ALBU_HUMAN	210	165	15	10	3
1358	1.44	0.07	1.73	**0.001**	− 1.21	0.32	Alpha-1-antitrypsin	A1AT_HUMAN	211	159	24	10	4
1478	− 1.12	0.66	1.42	0.15	− 1.59	0.12	Apolipoprotein A-I	APOA1_HUMAN	435	332	49	14	8
1483	− 1.05	0.82	1.44	**0.05**	− 1.51	**0.05**	Alpha-1-antitrypsin	A1AT_HUMAN	706	554	49	18	6
1484	− 1.08	0.74	1.40	0.14	− 1.51	0.13	Serum albumin	ALBU_HUMAN	474	410	18	13	5
F	1431	− 1.04	0.91	1.77	0.11	− 1.84	0.12	Vitamin D-binding protein	VTDB_HUMAN	292	228	16	9	5
1435	1.09	0.73	1.68	0.06	− 1.54	0.19	Vitamin D-binding protein	VTDB_HUMAN	345	309	24	8	4
1437	1.30	0.31	1.62	**0.03**	− 1.18	0.52	Transthyretin	TTHY_HUMAN	84	70	9	2	2
1438	1.34	0.22	1.59	**0.02**	− 1.25	0.39	Transthyretin	TTHY_HUMAN	102	81	18	3	2
1457	1.51	0.07	1.36	0.11	1.11	0.66	Haptoglobin	HPT_HUMAN	234	208	12	6	4
G	873	− 8.25	**0.05**	− 1.92	0.31	− 4.30	**0.03**	Vitamin D-binding protein	VTDB_HUMAN	365	295	23	12	5
875	− 1.13	0.83	4.42	0.07	− 5.01	0.06	Alpha-1-antitrypsin	A1AT_HUMAN	286	235	16	8	5
905	2.44	0.12	1.78	0.21	1.38	0.49	Apolipoprotein A-II	APOA2_HUMAN	61	17	21	3	3
906	1.56	0.56	1.75	0.28	− 0.12	0.86	Haptoglobin	HPT_HUMAN	56	42	6	2	1
1416	9.27	0.35	1.47	0.61	6.32	0.38	Apolipoprotein A-I	APOA1_HUMAN	126	s	16	4	3

Protein name, mass spectrometry data, fold differences between subgroups and their statistical significance are compiled for 36 of identified synovial fluid proteins. Spot trypsin digests were identified using matrix assisted laser desorption ionization (MALDI-TOF/TOF), correlated to compiled peptide data (Matrixscience). *p*-Values in bold highlight inter-subgroup comparisons which reached statistical significance (*p* < 0.05) by unpaired *t* test. Peptide ion sequence and peak lists can be found in a Supplement Table 1A–D. In a comparison between persistent oligoarticular patients and those who later exhibit disease extension, 23 of these proteins differed by at least 1.5 fold (labeled in Fig. 1).

## References

[1] Symmons D.P., Jones M., Osborne J., Sills J., Southwood T.R., Woo P. (1996). Pediatric rheumatology in the United Kingdom: data from the British Pediatric Rheumatology Group National Diagnostic Register. J Rheumatol.

[2] Petty R.E., Southwood T.R., Manners P., Baum J., Glass D.N., Goldenberg J. (2004). International League of Associations for Rheumatology. International League of Associations for Rheumatology classification of juvenile idiopathic arthritis: second revision, Edmonton, 2001. J Rheumatol.

[3] Flato B., Lien G., Smerdel A., Vinje O., Dale K., Johnston V. (2003). Prognostic factors in juvenile rheumatoid arthritis: a case–control study revealing early predictors and outcome after 14.9 years. J Rheumatol.

[4] Huemer C., Malleson P.N., Cabral D.A., Huemer M., Falger J., Zidek T. (2002). Patterns of joint involvement at onset differentiate oligoarticular juvenile psoriatic arthritis from pauciarticular juvenile rheumatoid arthritis. J Rheumatol.

[5] Woo P., Southwood T.R., Prieur A.M., Dore C.J., Grainger J., David J. (2000). Randomized, placebo-controlled, crossover trial of low-dose oral methotrexate in children with extended oligoarticular or systemic arthritis. Arthritis Rheum.

[6] Rosenkranz M.E., Wilson D.C., Marinov A.D., Decewicz A., Grof-Tisza P., Kirchner D. (2010). Synovial fluid proteins differentiate between the subtypes of juvenile idiopathic arthritis. Arthritis Rheum.

[7] Low J.M., Chauhan A.K., Gibson D.S., Zhu M., Chen S., Rooney M.E. (2009). Proteomic analysis of circulating immune complexes in juvenile idiopathic arthritis reveals disease-associated proteins. Proteomics Clin Appl.

[8] Gibson D.S., Blelock S., Brockbank S., Curry J., Healy A., McAllister C. (2006). Proteomic analysis of recurrent joint inflammation in juvenile idiopathic arthritis. J Proteome Res.

[9] Gibson D.S., Finnegan S., Jordan G., Scaife C., Brockbank S., Curry J. (2009). Stratification and monitoring of juvenile idiopathic arthritis patients by synovial proteome analysis. J Proteome Res.

[10] Hunter P.J., Nistala K., Jina N., Eddaoudi A., Thomson W., Hubank M. (2010). Biologic predictors of extension of oligoarticular juvenile idiopathic arthritis as determined from synovial fluid cellular composition and gene expression. Arthritis Rheum.

[11] Alavi A., Axford J.S. (2008). Sweet and sour: the impact of sugars on disease. Rheumatology (Oxford).

[12] Varki A. (2006). Nothing in glycobiology makes sense, except in the light of evolution. Cell.

[13] Wang X., Gu J., Ihara H., Miyoshi E., Honke K., Taniguchi N. (2006). Core fucosylation regulates epidermal growth factor receptor-mediated intracellular signaling. J Biol Chem.

[14] Poe J.C., Fujimoto Y., Hasegawa M., Haas K.M., Miller A.S., Sanford I.G. (2004). CD22 regulates B lymphocyte function in vivo through both ligand-dependent and ligand-independent mechanisms. Nat Immunol.

[15] Das T., Mandal C., Mandal C. (2004). Variations in binding characteristics of glycosylated human C-reactive proteins in different pathological conditions. Glycoconj J.

[16] Morgan R., Gao G., Pawling J., Dennis J.W., Demetriou M., Li B. (2004). N-acetylglucosaminyltransferase V (Mgat5)-mediated N-glycosylation negatively regulates Th1 cytokine production by T cells. J Immunol.

[17] Pappu B.P., Shrikant P.A. (2004). Alteration of cell surface sialylation regulates antigen-induced naive CD8 + T cell responses. J Immunol.

[18] Ferens-Sieczkowska M., Kossowska B., Gancarz R., Dudzik D., Knas M., Popko J. (2007). Fucosylation in synovial fluid as a novel clinical marker for differentiating joint diseases—a preliminary study. Clin Exp Rheumatol.

[19] Higai K., Aoki Y., Azuma Y., Matsumoto K. (2005). Glycosylation of site-specific glycans of alpha1-acid glycoprotein and alterations in acute and chronic inflammation. Biochim Biophys Acta.

[20] Raghav S.K., Gupta B., Agrawal C., Saroha A., Das R.H., Chaturvedi V.P. (2006). Altered expression and glycosylation of plasma proteins in rheumatoid arthritis. Glycoconj J.

[21] Gindzienska-Sieskiewicz E., Klimiuk P.A., Kisiel D.G., Gindzienski A., Sierakowski S. (2007). The changes in monosaccharide composition of immunoglobulin G in the course of rheumatoid arthritis. Clin Rheumatol.

[22] Omtvedt L.A., Royle L., Husby G., Sletten K., Radcliffe C.M., Harvey D.J. (2006). Glycan analysis of monoclonal antibodies secreted in deposition disorders indicates that subsets of plasma cells differentially process IgG glycans. Arthritis Rheum.

[23] Gibson D.S., Blelock S., Curry J., Finnegan S., Healy A., Scaife C. (2009). Comparative analysis of synovial fluid and plasma proteomes in juvenile arthritis—proteomic patterns of joint inflammation in early stage disease. J Proteomics.

[24] Sinz A., Bantscheff M., Mikkat S., Ringel B., Drynda S., Kekow J. (2002). Mass spectrometric proteome analyses of synovial fluids and plasmas from patients suffering from rheumatoid arthritis and comparison to reactive arthritis or osteoarthritis. Electrophoresis.

[25] Ling X.B., Park J.L., Carroll T., Nguyen K.D., Lau K., Macaubas C. (2010). Plasma profiles in active systemic juvenile idiopathic arthritis: biomarkers and biological implications. Proteomics.

[26] Zhang J., Habiel D.M., Ramadass M., Kew R.R. (2010). Identification of two distinct cell binding sequences in the vitamin D binding protein. Biochim Biophys Acta.

[27] Daiger S.P., Schanfield M.S., Cavalli-Sforza L.L. (1975). Group-specific component (Gc) proteins bind vitamin D and 25-hydroxyvitamin D. Proc Natl Acad Sci U S A.

[28] Ena J.M., Esteban C., Perez M.D., Uriel J., Calvo M. (1989). Fatty acids bound to vitamin D-binding protein (DBP) from human and bovine sera. Biochem Int.

[29] Kanda S., Mochizuki Y., Miyata Y., Kanetake H., Yamamoto N. (2002). Effects of vitamin D(3)-binding protein-derived macrophage activating factor (GcMAF) on angiogenesis. J Natl Cancer Inst.

[30] Speeckaert M., Huang G., Delanghe J.R., Taes Y.E. (2006). Biological and clinical aspects of the vitamin D binding protein (Gc-globulin) and its polymorphism. Clin Chim Acta.

[31] Braun A., Kofler A., Morawietz S., Cleve H. (1993). Sequence and organization of the human vitamin D-binding protein gene. Biochim Biophys Acta.

[32] Ravnsborg T., Olsen D.T., Thysen A.H., Christiansen M., Houen G., Hojrup P. (2010). The glycosylation and characterization of the candidate Gc macrophage activating factor. Biochim Biophys Acta.

[33] Yamamoto N., Homma S. (1991). Vitamin D3 binding protein (group-specific component) is a precursor for the macrophage-activating signal factor from lysophosphatidylcholine-treated lymphocytes. Proc Natl Acad Sci U S A.

[34] Borges C.R., Jarvis J.W., Oran P.E., Nelson R.W. (2008). Population studies of vitamin D binding protein microheterogeneity by mass spectrometry lead to characterization of its genotype-dependent O-glycosylation patterns. J Proteome Res.

[35] Chen Y.C., Wang P.W., Pan T.L., Bazylak G., Shen J.J. (2010). Proteomic analysis of plasma to reveal the impact of short-term etanercept therapy in pediatric patients with enthesitis-related arthritis: a case report. Comb Chem High Throughput Screen.

[36] Takeuchi T., Kotani T., Nakanishi T., Tabushi-Matsumura Y., Takubo T., Makino S. (2010). Proteomic analysis of changes in the serum protein profile by anti-TNF-alpha therapy]. Rinsho Byori.

[37] Stoop M.P., Singh V., Dekker L.J., Titulaer M.K., Stingl C., Burgers P.C. (2010). Proteomics comparison of cerebrospinal fluid of relapsing remitting and primary progressive multiple sclerosis. PLoS One.

[38] Rozanov D.V., Savinov A.Y., Golubkov V.S., Postnova T.I., Remacle A., Tomlinson S. (2004). Cellular membrane type-1 matrix metalloproteinase (MT1-MMP) cleaves C3b, an essential component of the complement system. J Biol Chem.

[39] Miller J.J., Olds L.C., Huene D.B. (1986). Complement activation products and factors influencing phagocyte migration in synovial fluids from children with chronic arthritis. Clin Exp Rheumatol.

[40] Mollnes T.E., Paus A. (1986). Complement activation in synovial fluid and tissue from patients with juvenile rheumatoid arthritis. Arthritis Rheum.

