# Supplementary material for: Vitamin D binding protein isoforms as candidate predictors of disease extension in childhood arthritis
Source: J Proteomics. 2012 Sep 18;75(17):5479–92. doi: 10.1016/j.jprot.2012.06.024 (PMC3443749; doi:10.1016/j.jprot.2012.06.024)
Supplement: Supplementary file 1 — Supplementary materials. [file mmc1.pdf]

Cluster	Spot No.	Protein Name	Accession Number	Verification CID ions	Peptide position in protein sequence: Start-End	Observed mass	Ions Score	Amino Acid Sequence	Variable Modification Carbymidomethyl Cysteine (C), Oxidation Methionine (M)
A	428	Complement factor B	CFAB_HUMAN	1	183-193	1366.5308	62	LEDSVTYHCSR	C
A	529	Ig gamma-2 chain C region	IGHG2_HUMAN	3	206-213	824.5599	22	GLPAPIEK	
					224-234	1286.7853	62	EPQVYTLPPSR	
					17-30	1423.8301	62	STSESTAALGCLVK	C
A	530	Ig kappa chain C region	IGKC_HUMAN	2	83-99	1875.8892	80	VYACEVTHQGLSSPVTK	C
					42-61	2135.9272	116	VDNALQSGNSQESVTEQ	
A	750	Haptoglobin	HPT_HUMAN	6	271-277	809.4557	44	DYAEVGR	
					171-178	920.5434	27	GSFPWQAK	
					278-286	980.585	35	VGYVSGWGR	
					312-321	1146.653	40	HYEGSTVPEK	
					380-391	1345.7632	58	SCAVAEYGVYVK	C
					298-311	1723.9751	8	YVMLPVADQDQCIR	C,M
A	861	Haptoglobin	HPT_HUMAN	7	271-277	809.3728	40	DYAEVGR	
					171-178	920.447	33	GSFPWQAK	
					278-286	980.4863	26	VGYVSGWGR	
					312-321	1146.5382	53	HYEGSTVPEK	
					380-391	1345.639	92	SCAVAEYGVYVK	C
					298-311	1707.8241	46	YVMLPVADQDQCIR	C
					298-311	1723.8132	15	YVMLPVADQDQCIR	C,M
A	1346	Apolipoprotein A-IV	APOA4_HUMAN	2	135-143	1104.6632	29	LEPYADQLR	
					144-154	1287.7764	21	TQVNTQAEQLR	
B	228	Alpha-1-antitrypsin	A1AT_HUMAN	4	299-305	922.45	41	FLENEDR	
					325-334	1015.642	25	SVLGQLGITK	
					299-306	1078.5509	16	FLENEDRR	
					284-298	1804.0233	35	LQHLENELTHDIITK	
B	1302	Alpha-1-antitrypsin	A1AT_HUMAN	3	299-305	922.5031	40	FLENEDR	
					299-306	1078.6161	38	FLENEDRR	
					226-241	1892.0131	111	DTEEEDFHVDQVTTVK	
B	1303	Alpha-1-antitrypsin	A1AT_HUMAN	5	218-225	1090.5095	15	WERPFEVK	
					248-257	1263.5339	24	LGMFNIQHCK	C,M
					216-225	1275.6222	16	GKWERPFEVK	
					35-49	1779.6857	99	TDTSHHDQDHPTFNK	
					226-241	1891.7744	95	DTEEEDFHVDQVTTVK	
B	1375	Serum albumin	ALBU_HUMAN	5	427-434	960.5436	39	FQNALLVR	
					287-298	1443.6056	48	YICENQDSISSK	C
					439-452	1511.8086	19	VPQVSTPTLVEVSR	
					438-452	1639.9021	21	KVPQVSTPTLVEVSR	
					265-281	2086.7874	51	VHTECCHGDLLECADDR	C
B	1376	Serum albumin	ALBU_HUMAN	7	427-434	960.5309	34	FQNALLVR	
					287 298	1443.5924	49	YICENQDSISSK	C
					439-452	1511.797	65	VPQVSTPTLVEVSR	
					438-452	1639.8843	59	KVPQVSTPTLVEVSR	
					509-524	1910.8721	36	RPCFSALEVDETYVPK	C
					265-281	2086.7761	77	VHTECCHGDLLECADDR	C
C	196	Transthyretin	TTHY_HUMAN	4	42-54	1366.8008	60	GSPAINVAVHVFR	
					56-68	1394.6648	99	AADDTWEPFASGK	
					55-68	1522.7604	113	KAADDTWEPFASGK	
					69-90	2455.2195	144	TSESGELHGLTTEEEFVEGIYK	
C	1252	Vitamin D-binding protein	VTDB_HUMAN	5	346-352	915.3862	33	YTFELSR	
					296-303	1115.3375	36	FEDCCQEK	C
					219-229	1275.4784	74	VCSQYAAYGEK	C
					353-363	1326.6614	6	RTHLPEVFLSK	
					403-419	2054.7354	74	GQELCADYSENTFTEYK	C
C	1253	Vitamin D-binding protein	VTDB_HUMAN	10	346-352	915.5214	33	YTFELSR	
					296-303	1115.4995	33	FEDCCQEK	C
					354-363	1170.7352	45	THLPEVFLSK	
					219-229	1275.662	69	VCSQYAAYGEK	C
					353-363	1326.8523	57	RTHLPEVFLSK	
					51-65	1695.0393	69	KFPSGTFEQVSQLVK	
					403-419	2055.0227	124	GQELCADYSENTFTEYK	C
					95-114	2265.1157	35	SCESNSPFPVHPGTAECCTK	C
					66-87	2518.2063	53	EVVSLTEACCAEGADPDCYDTR	C
					128-149	2707.4092	122	HQPQEFPTYVEPTNDEICEAFR	C
C	1265	Haptoglobin	HPT_HUMAN	7	60-71	1311.649	92	TEGDGVYTLNDK	
					142-153	1387.7151	75	LPECEAVCGKPK	C
					119-131	1439.7128	94	TEGDGVYTLNNEK	
					58-71	1580.8433	23	LRTEGDGVYTLNDK	
					117-131	1708.9108	74	LRTEGDGVYTLNNEK	
					137-153	1857.9916	59	AVGDKLPECEAVCGKPK	C
					83-108	2962.488	99	LPECEADDGCPKPPEIAHGYVEHSVR	C
D	652	Haptoglobin	HPT_HUMAN	3	142-153	1387.6378	10	LPECEAVCGKPK	C
					119-131	1439.64	92	TEGDGVYTLNNEK	
					117-131	1708.8262	4	LRTEGDGVYTLNNEK	
D	781	Haptoglobin	HPT_PONAB	2	112-119	920.5518	46	GSFPWQAK	
					321-332	1345.7795	100	SCAVAEYGVYVK	C

D	876	Alpha-1B-glycoprotein	A1BG_HUMAN	1	79-90	1372.8027	80	HQFLLTGDTQGR	
E	175	Serotransferrin	TRFE_HUMAN	9	62-69	997.5654	34	ASYLDCIR	C
					61-69	1125.6753	29	KASYLDCIR	C
					123-132	1195.6615	44	DSGFQMNQLR	
					123-132	1211.6603	15	DSGFQMNQLR	M
					454-464	1249.7167	36	SASDLTWDNLK	
					300-310	1276.7499	38	EFQLFSSPHGK	
					259-273	1690.0062	70	DCHLAQVPSHTVVAR	C
					385-399	1725.9276	107	IECVSAETTEDCIAK	C
					347-362	1817.9702	69	EGTCPEAPTDECKPVK	C
E	178	Serotransferrin	TRFE_HUMAN	11	62-69	997.4507	41	ASYLDCIR	C
					61-69	1125.5513	11	KASYLDCIR	C
					123-132	1195.5303	59	DSGFQMNQLR	
					123-132	1211.5265	26	DSGFQMNQLR	M
					226-236	1273.6316	77	HSTIFENLANK	
					122-132	1323.623	23	KDSGFQMNQLR	
					47-60	1415.6963	53	SVIPSDGPSVACVK	C
					240-251	1539.6871	47	DQYELLCLDNTR	C
					108-121	1629.795	58	EDPQTFYYAVAVVK	
					259-273	1689.825	68	DCHLAQVPSHTVVAR	C
					237-251	1881.853	34	ADRDQYELLCLDNTR	C
E	191	Serotransferrin	TRFE_HUMAN	10	62-69	997.4554	26	ASYLDCIR	C
					123-132	1195.5337	52	DSGFQMNQLR	
					123-132	1211.5297	11	DSGFQMNQLR	M
					300-310	1276.6113	32	EFQLFSSPHGK	
					122-132	1323.6249	9	KDSGFQMNQLR	
					240-251	1539.689	43	DQYELLCLDNTR	C
					108-121	1629.7931	57	EDPQTFYYAVAVVK	
					259-273	1689.8243	53	DCHLAQVPSHTVVAR	C
					237-251	1881.8594	40	ADRDQYELLCLDNTR	C
					252-273	2549.2634	52	KPVDEYKDCHLAQVPSHTVVAR	C
E	557	Serum albumin	ALBU_HUMAN	3	427-434	960.615	42	FQNALLVR	
					287-298	1443.7166	46	YICENQDSISSK	C
					265-281	2086.9465	77	VHTECCHGDLLECADDR	C
E	1358	Alpha-1-antitrypsin	A1AT_HUMAN	4	299-305	922.4437	30	FLENEDR	
					325-334	1015.6359	29	SVLGQLGITK	
					299-306	1078.5486	45	FLENEDRR	
					315-324	1110.6322	55	LSITGTYDLK	
E	1478	Apolipoprotein A-I	APOA1_HUMAN	8	213-219	831.4572	24	LAEYHAK	
					148-155	873.4694	20	AELQEGAR	
					231-239	1012.6069	48	AKPALEDLR	
					165-173	1031.552	26	LSPLGEEMR	
					165-173	1047.5607	10	LSPLGEEMR	M
					121-130	1252.6576	50	VQPYLDDFQK	
					185-195	1301.6859	47	THLAPYSDELR	
					52-64	1400.7137	117	DYVSQFEGSALGK	
E	1483	Alpha-1-antitrypsin	A1AT_HUMAN	6	284-298	1803.9786	101	LQHLENELTHDIITK	
					390-404	1871.9985	50	FNKPFVFLMIEQNTK	M
					226-241	1891.8762	110	DTEEEDFHVDQVTTVK	
					161-178	2057.9683	166	LYHSEAFTVNFGDTEEAK	
					161-179	2186.0681	105	LYHSEAFTVNFGDTEEAKK	
					368-389	2291.1711	23	GTEAAGAMFLEAIPMSIPPEVK	M
E	1484	Serum albumin	ALBU_HUMAN	5	427-434	960.5902	58	FQNALLVR	
					287-298	1443.6788	77	YICENQDSISSK	C
					439-452	1511.8859	72	VPQVSTPTLVEVSR	
					438-452	1639.9819	75	KVPQVSTPTLVEVSR	
					265-281	2086.887	129	VHTECCHGDLLECADDR	C
F	1431	Vitamin D-binding protein	VTDB_HUMAN	5	346-352	915.3845	26	YTFELSR	
					354-363	1170.5615	65	THLPEVFLSK	
					219-229	1275.474	43	VCSQYAAYGEK	C
					353-363	1326.6566	41	RTHLPEVFLSK	
					403-419	2054.7148	53	GQELCADYSENTFTEYK	C
F	1435	Vitamin D-binding protein	VTDB_HUMAN	5	346-352	915.4045	34	YTFELSR	
					296-303	1115.3606	52	FEDCCQEK	C
					219-229	1275.5028	68	VCSQYAAYGEK	C
					353-363	1326.6912	35	RTHLPEVFLSK	
					403-419	2054.7705	106	GQELCADYSENTFTEYK	C
F	1437	Transthyretin	TTHY_HUMAN	2	56-68	1394.6556	34	AADDTWEPFASGK	
					55-68	1522.751	35	KAADDTWEPFASGK	
F	1438	Transthyretin	TTHY_HUMAN	2	56-68	1394.6677	50	AADDTWEPFASGK	
					55-68	1522.7581	31	KAADDTWEPFASGK	
F	1457	Haptoglobin	HPT_HUMAN	4	142-153	1387.7249	47	LPECEAVCGKPK	C
					119-131	1439.7184	78	TEGDGVYTLNNEK	
					117-131	1708.9155	40	LRTEGDGVYTLNNEK	
					137-153	1857.9945	44	AVGDKLPECEAVCGKPK	C
G	873	Vitamin D-binding protein	VTDB_HUMAN	4	31 37	817.4254	30	EFSHLGK	
					95 114	2264.9573	92	SCESNSPFPVHPGTAECCTK	C
					66 87	2518.0388	140	EVVSLTEACCAEGADPDCYDTR	C
					128 149	2707.2324	46	HQPQEFPTYVEPTNDEICEAFR	C
G	875	Alpha-1-antitrypsin	A1AT_HUMAN	5	218-225	1090.4999	20	WERPFEVK	
					248-257	1263.5128	30	LGMFNIQHCK	C,M

					216-225	1275.6035	1	GKWERPFEVK
					35-49	1779.6571	96	TDTSHHDQDHPTFNK
					226-241	1891.7438	90	DTEEEDFHVDQVTTVK
G	905	Apolipoprotein A-II	APOA2_HUMAN	3	54-62	972.5055	10	SPELQAEAK
					68-77	1156.6864	17	SKEQLTPLIK
					52-62	1199.675	7	VKSPELQAEAK
G	906	Haptoglobin	HPT_HUMAN	1	60-72	1439.6349	43	TEGDGVYTLNDKK
G	1416	Apolipoprotein A-I	APOA1_HUMAN	3	121-130	1252.668	26	VQPYLDDFQK
					185-195	1301.7043	29	THLAPYSDELR
					52-64	1400.725	45	DYVSQFEGSALGK

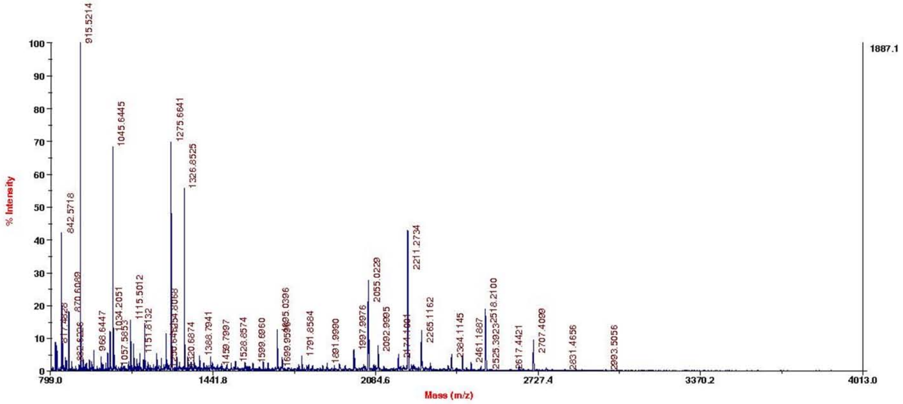

Spot 1253 Vit. D BP (Est. 51.1 KDa)

1 MKRVLVLLLA VAFGHALERG RDYEKNKVCK **EFSHLGK**EDF TSLSLVLYSR
51 **KFPSGTFEQV SQLVKEVVSL TEACCAEGAD PDCYDTR**TSA LSAK**SCESNS**
101 **PFPVHPGTAE CCTK**EGLERK LCMAALK**HQP QEFPTYVEPT NDEICEAFR**K
151 DPKEYANQFM WEYSTNYGQA PLSLLVSYTK SYLSMVGSCC TSASPTVCFL
201 KERLQLK**HLS LLTTLSNRVC SQYAAYGEK**K SRLSNLIKLA QKVPTADLED
251 VLPLAEDITN ILSKCCESAS EDCMAKELPE HTVKLCDNLS TKNSK**FEDCC**
301 **QEK**TAMDVFV CTYFMPAAQL PELPDVELPT NKDVCDPGNT K**VMDKYTFEL**
351 **SRRTHLPEVF LSK**VLEPTLK **SLGECCDVED STTCFNAK**GP LLKKELSSFI
401 DK**GQELCADY SENTFTEYKK** KLAERLKAKL PDATPKELAK LVNKRSDFAS
451 NCCSINSPPL YCDSEIDAEL KNIL

A

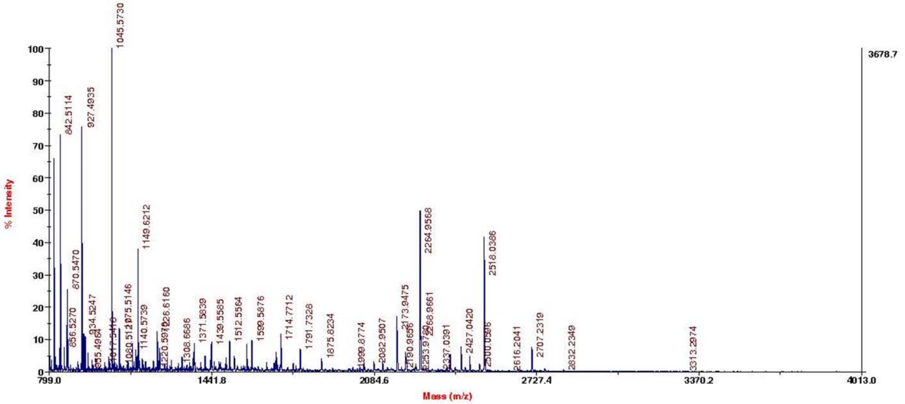

Spot 1435 Vit. D BP (Est. 51.1 KDa)

1 MKRVLVLLLA VAFGHALERG RDYEKNKVCK **EFSHLGKEDF TSLSLVLYSR**
51 **KFPSGTFEQV SQLVKEVVSL TEACCAEGAD PDCYDTR**TSA LSAK**SCESNS**
101 **PFPVHPGTAE CCTK**EGLER**K LCMAALKHQP QEFPTYVEPT NDEICEAFR**K
151 DPKEYANQFM WEYSTNYGQA PLSLLVSYTK SYLSMVGSCC TSASPTVCFL
201 KERLQLKHLS LLTTLSNRVC SQYAAYGEKK SRLSNLIKLA QKVPTADLED
251 VLPLAEDITN ILSKCCESAS EDCMAKELPE HTVKLCDNLS TKNSKFEDCC
301 QEKTAMDVFV CTYFMPAAQL PELPDVELPT NKDVCDPGNT KVMDKYTFEL
351 SRRTHLPEVF LSKVLEPTLK SLGECCDVED STTCFNAKGP LLKKELSSFI
401 DKGQELCADY SENTFTEYKK KLAERLK**AKL PDATPK**ELAK LVNKRSDFAS
451 NCCSINSPPL YCDSEIDAEL KNIL

B

Supplement Figure 1
